# Supplementary material for: Premature birth changes wiring constraints in neonatal structural brain networks
Source: Nat Commun. 2025 Jan 8;16:490. doi: 10.1038/s41467-024-55178-x (PMC11711473; doi:10.1038/s41467-024-55178-x)
Supplement: Supplementary file 1 — Supplementary Information [file 41467_2024_55178_MOESM1_ESM.pdf]

Supplementary Information:

Premature birth changes wiring constraints in neonatal structural brain networks

Alexa Mousley[1*], Danyal Akarca[1,2,3] & Duncan E. Astle[1,4]

[1]MRC Cognition and Brain Sciences Unit, University of Cambridge, Cambridge, UK

[2]Department of Electrical and Electronic Engineering, Imperial College London, London, UK

[3]Imperial-X, Imperial College London, London, UK

[4]Department of Psychiatry, University of Cambridge, Cambridge, UK

*Corresponding author: Alexa Mousley

Email: alexa.mousley@mrc-cbu.cam.ac.uk

Address: 15 Chaucer Road, Cambridge, CB2 7EF UK

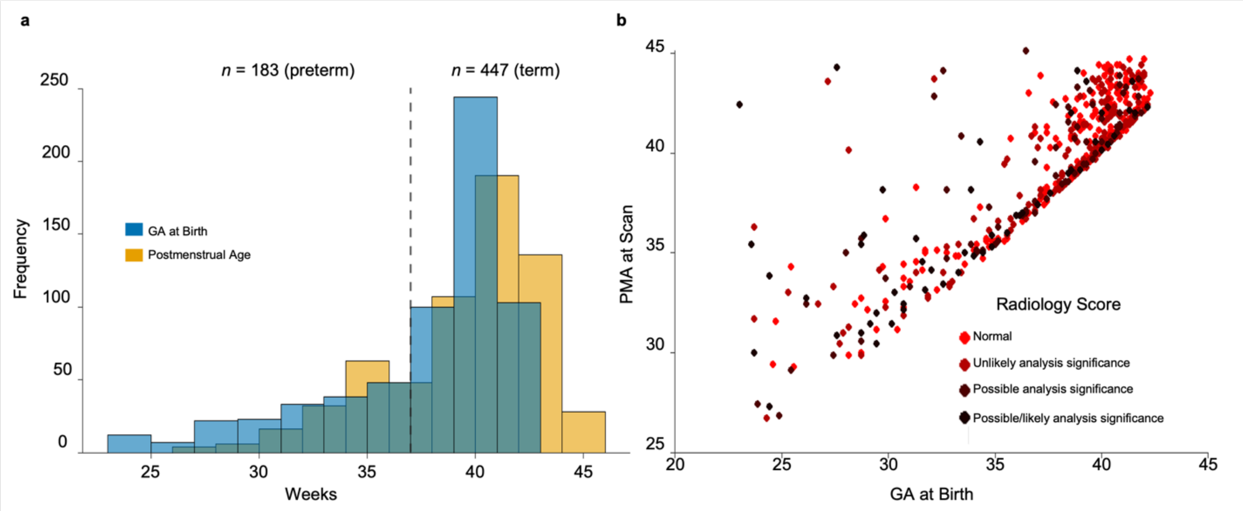

Supplementary Figure 1. Participant details. (a) Histogram depicting the distribution of GA at birth and PMAs in the sample. **(b)** Participants' GA at birth was plotted against their PMA at scan, grouped by the dHCP radiology score from their structural images.

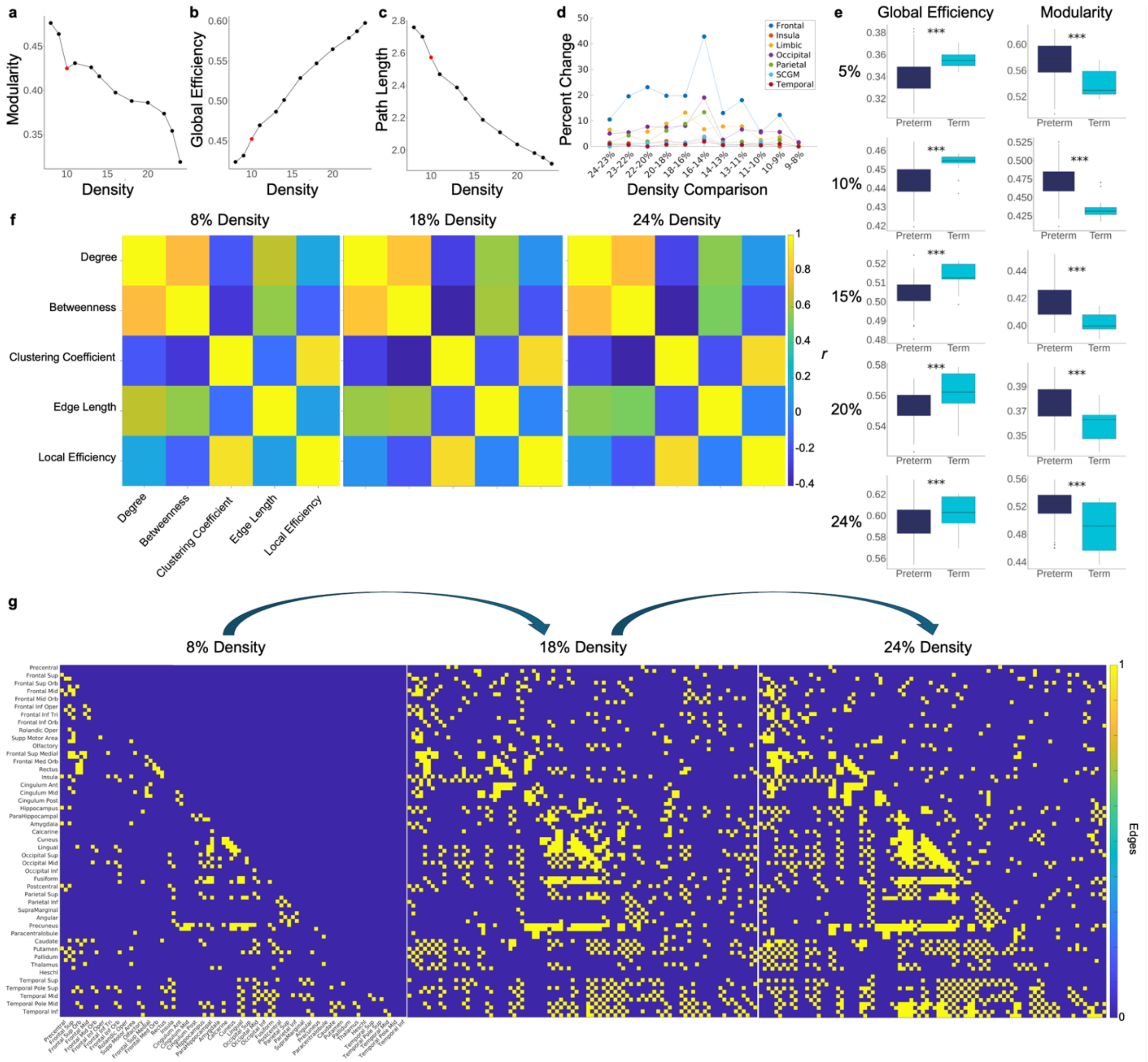

Supplementary Figure 2. Exploration of multiple density networks. Across consensus networks, **(a)** modularity decreases**, (b)** global efficiency increases, and **(c)** characteristic path length decreases with increasingly dense connectomes. **(d)** Comparisons of consecutive consensus networks, indicating the distribution of new connections across 7 lobes (indicated as a % change from the sparser to dense network). **(e)** In the propensity-matched analysis, preterm infants at term-equivalent age have significantly less efficient, more modular networks in compared to term infants across 5-24% average density networks (global efficiency: 5% $t(189.15) = -10.76$, $p < 2.20 \times 10^{-16}$; 10% $t(190.91) = -8.28$, $p = 2.07 \times 10^{-14}$; 15% $t(237.49) = -9.66$, $p < 2.20 \times 10^{-16}$; 20% $t(220.23) = -5.76$, $p = 2.75 \times 10^{-8}$; 24% $t(237.75) = -4.01$, $p = 8.13 \times 10^{-5}$; modularity: 5% $t(221.97) = 11.60$, $p < 2.20 \times 10^{-16}$; 10% $t(224.80) = 14.31$, $p < 2.20 \times 10^{-16}$; 15% $t(193.12) = 12.06$, $p < 2.20 \times 10^{-16}$; 20% $t(235.52) = 9.35$, $p < 2.20 \times 10^{-16}$; 24% $t(197.76) = 8.65$, $p = 1.81 \times 10^{-15}$). **(f)** Topological fingerprints demonstrating the correlation between local organizational measures for the 8%, 18% and 24% density networks. **(g)** Connectome matrices representing the 8%, 18% and 24% networks. Here, the lower triangle is the matrix and for 18% and 24% networks the upper triangle is only comprised of connections that are present in that network that weren't present in the previous network (8-18% and 18-24% comparisons).

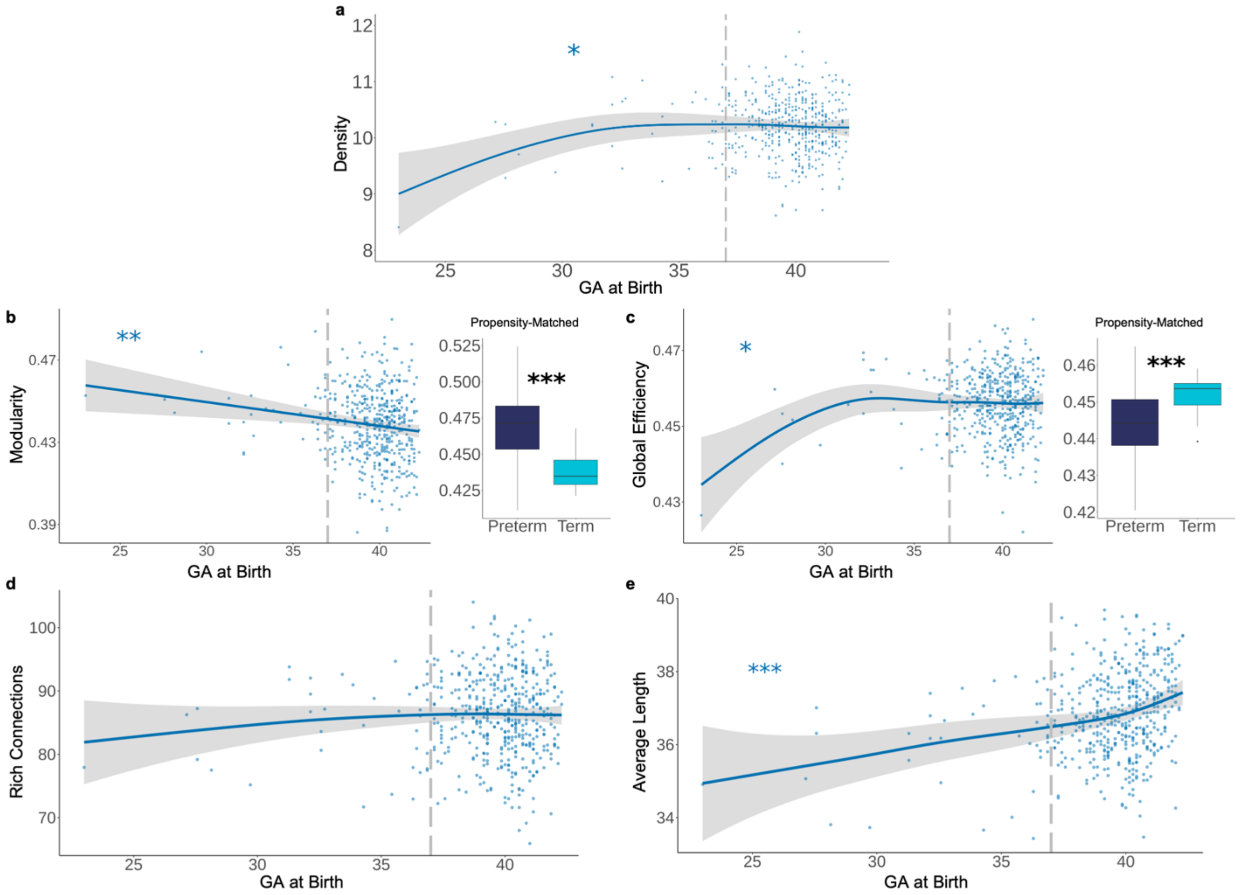

Supplementary Figure 3. Term-equivalent age topological analysis. (a) Density significantly increases across GA at birth. **(b)** Modularity significantly decreases across GA at birth and propensity-matched analysis shows that preterm infants have significantly higher modularity compared to term infants. **(c)** Global efficiency significantly increases across GA at birth and propensity-matched shows preterm infants have significantly lower global efficiency compared to term infants. **(d)** There is no significant change in the number of rich club connections, however **(e)** the average length of rich club connections significantly increases across GA at birth. The shaded area indicates 95% confidence intervals. The grey dotted line indicates the cut-off for term birth (37 weeks GA or later is term-born). *** indicates $p < 0.001$, ** indicates $p < 0.01$, * indicates $p < 0.05$.

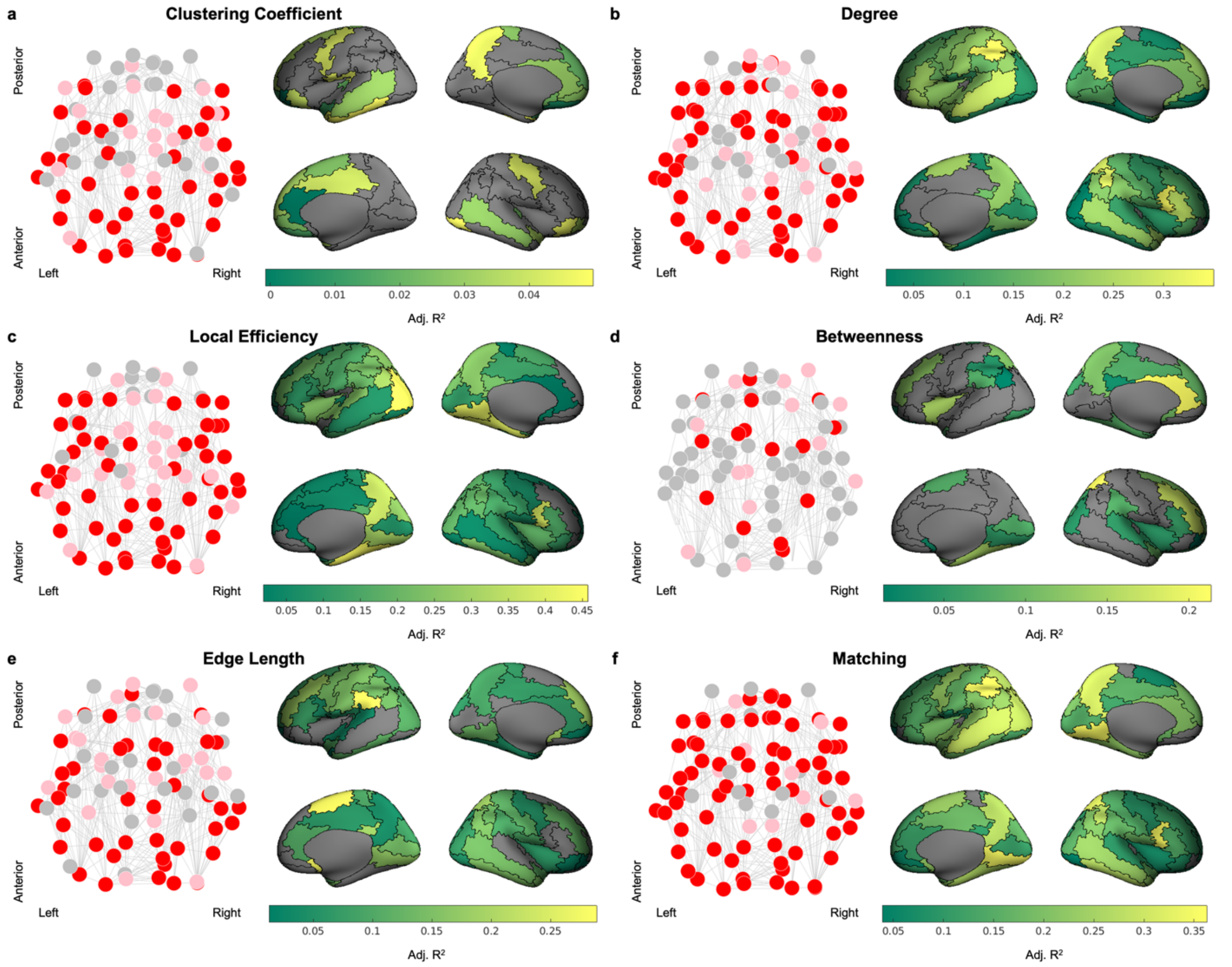

Supplementary Figure 4. Local measures changed significantly across PMA. Significant effects of PMA were found for **(a)** clustering coefficient, **(b)** degree, **(c)** local efficiency, **(d)** betweenness, **(e)** edge length (mm), and **(f)** matching. Red indicates $p < 0.001$ and pink indicates $p < 0.05$. Surface plots display the adjusted R^2 from GAMs. All node and surface plots are adapted from AAL90 atlas in Shi F, et al. (2011) Infant Brain Atlases from Neonates to 1- and 2-Year-Olds. PLoS ONE 6(4): e18746. doi:10.1371/journal.pone.0018746.

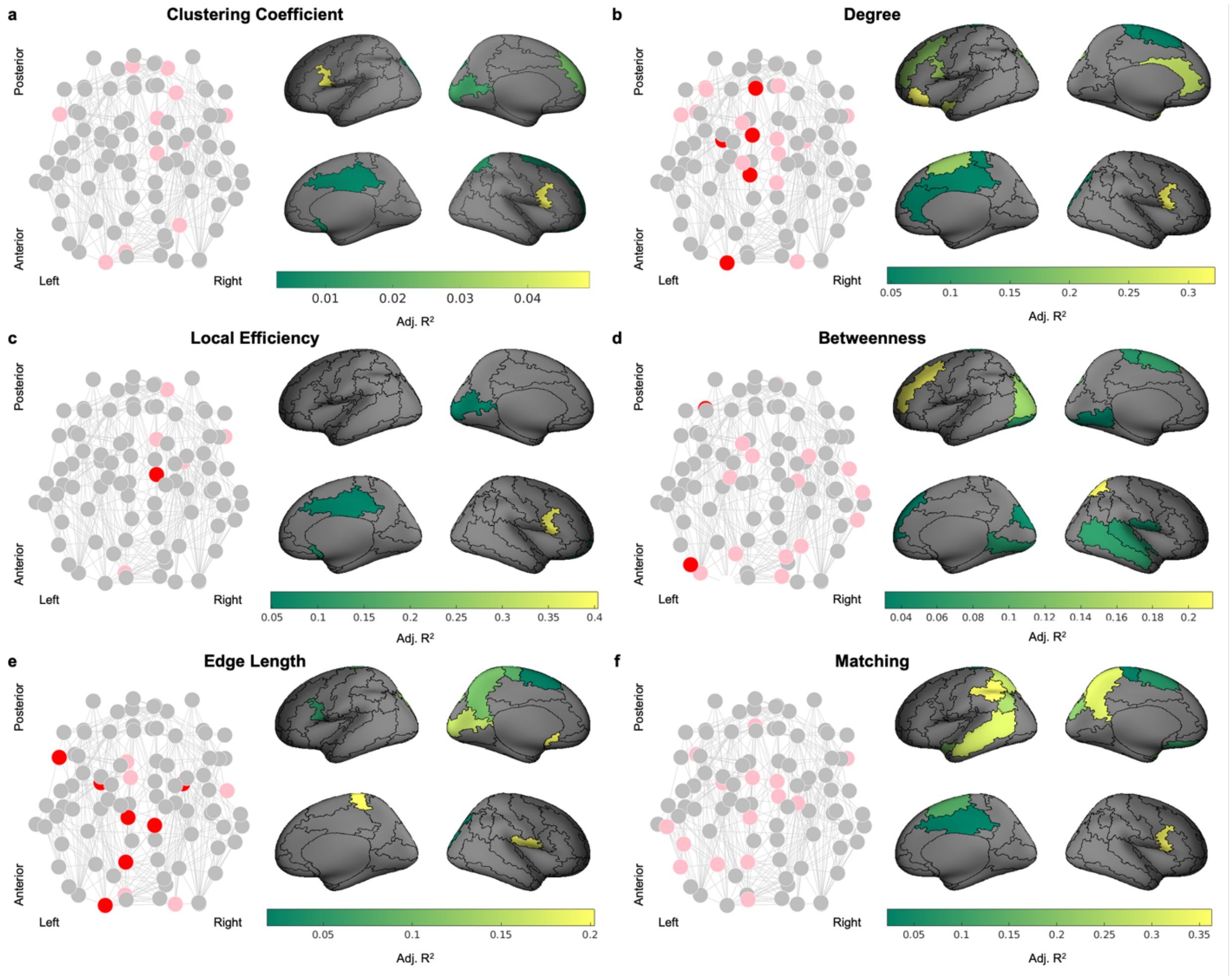

Supplementary Figure 5. Local measures changed significantly across GA at birth. Significant effects of GA at birth were found for **(a)** clustering coefficient, **(b)** degree, **(c)** local efficiency, **(d)** betweenness, **(e)** edge length (mm), and **(f)** matching. Red indicates $p < 0.001$ and pink indicates $p < 0.05$. Surface plots display the adjusted R^2 from GAMs. All node and surface plots are adapted from AAL90 atlas in Shi F, et al. (2011) Infant Brain Atlases from Neonates to 1- and 2-Year-Olds. PLoS ONE 6(4): e18746. doi:10.1371/ journal.pone.0018746.

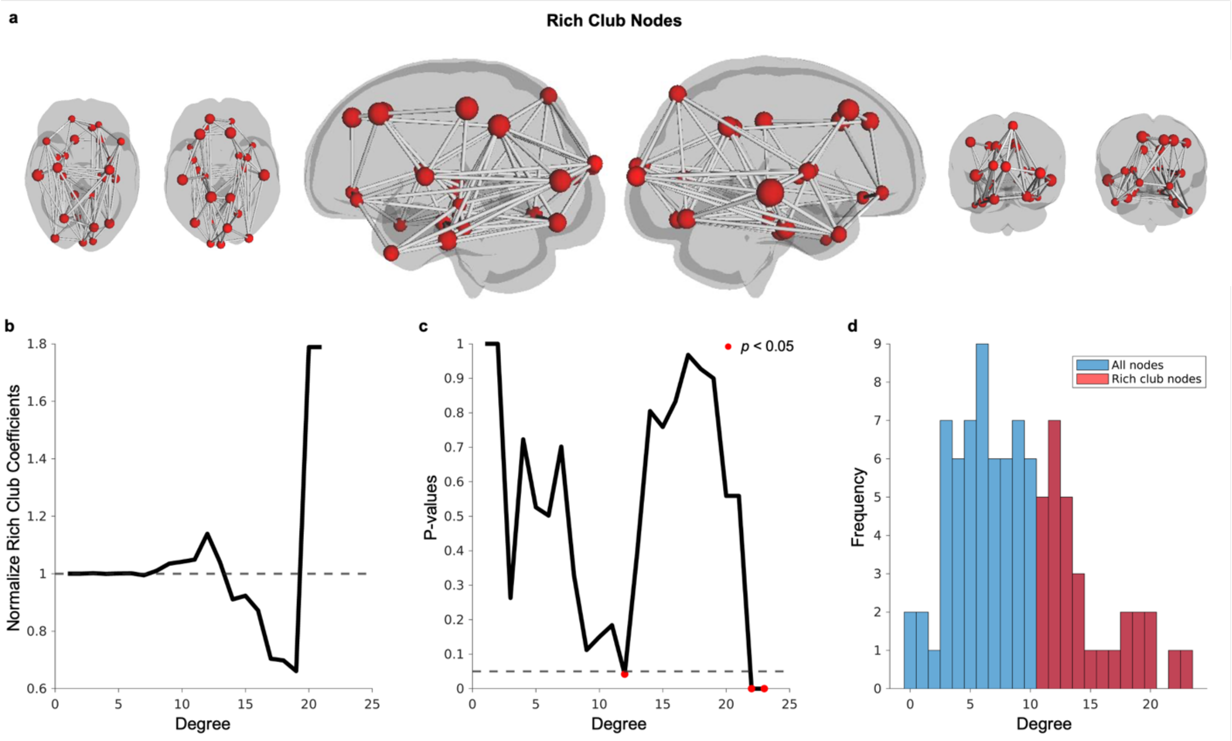

Supplementary Figure 6. The definition of rich club nodes. (a) Visualization of the 27 rich club nodes and connections in the consensus network. The node connectivity plots are adapted from AAL90 atlas in Shi F, et al. (2011) Infant Brain Atlases from Neonates to 1- and 2-Year-Olds. PLoS ONE 6(4): e18746. doi:10.1371/ journal.pone.0018746. **(b)** The normalized rich club coefficients, with two distinct instances of coefficients > 1. **(c)** The comparison to null models demonstrates two instances where the consensus network rich club coefficients are significantly greater than would be expected at random (red dots). **(d)** Histogram of the distribution of degree in the consensus network.

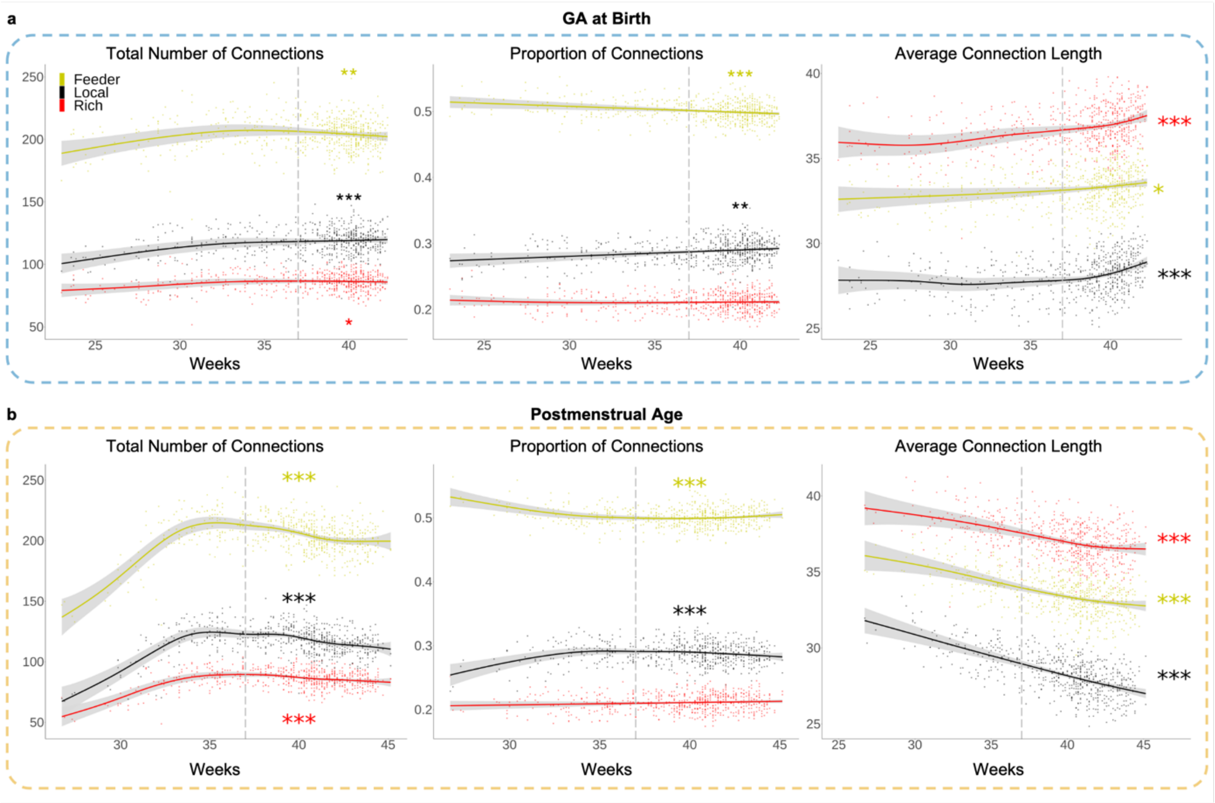

Supplementary Figure 7. Counts, proportions, and lengths of all connection types in variable-density networks across PMA and GA at birth. (a) Across GA at birth, the total number of rich, feeder and local connections significantly increased ($F_{rich\ connections,GA\ at\ birth} = 3.05$, estimated df = 2.69, $p = 0.022$; $F_{feeder\ connections,GA\ at\ birth} = 3.55$, estimated df = 3.04, $p = 1.87 \times 10^{-3}$; $F_{local\ connections,GA\ at\ birth} = 6.94$, estimated df = 2.79, $p = 6.73 \times 10^{-5}$). However, proportional to the total number of connections in the network, feeder connections decreased ($F_{feeder\ proportion,GA\ at\ birth} = 11.48$, estimated df = 1.00, $p = 7.42 \times 10^{-4}$), local connections increased ($F_{local\ proportion,GA\ at\ birth} = 8.98$, estimated df = 1.00, $p = 2.82 \times 10^{-3}$) and rich club connections do not significantly change ($p = 0.623$). The average length of connections increased for all connection types across GA at birth ($F_{rich\ length,GA\ at\ birth} = 5.37$, estimated df = 3.82, $p = 1.06 \times 10^{-4}$; $F_{feeder\ length,GA\ at\ birth} = 3.00$, estimated df = 2.03, $p = 0.031$; $F_{local\ length,GA\ at\ birth} = 6.36$, estimated df = 4.25, $p = 7.79 \times 10^{-6}$). **(b)** Across PMA, the total number of rich, feeder and local connections significantly increased ($F_{rich\ connections,PMA} = 16.62$, estimated df = 5.92, $p = 2.00 \times 10^{-16}$; $F_{feeder\ connections,PMA} = 23.02$, estimated df = 7.01, $p = 2.00 \times 10^{-16}$; $F_{local\ connections,PMA} = 20.57$, estimated df = 7.16, $p = 2.00 \times 10^{-16}$). However, proportional to the total number of connections in the network, feeder connections decreased ($F_{feeder\ proportion,PMA} = 6.63$, estimated df = 3.89, $p = 7.01 \times 10^{-6}$), local connections increased ($F_{local\ proportion,PMA} = 6.57$, estimated df = 4.24, $p = 3.71 \times 10^{-6}$) and rich club connections do not significantly change ($p = 0.171$). The average length of connections decreased for all connection types across PMA ($F_{rich\ length,PMA} = 11.92$, estimated df = 3.04, $p = 2.00 \times 10^{-16}$; $F_{feeder\ length,PMA} = 19.15$, estimated df = 2.77, $p = 2.00 \times 10^{-16}$; $F_{local\ length,PMA} = 59.70$, estimated df = 1.85, $p = 2.00 \times 10^{-16}$). The shaded area indicates 95% confidence intervals. The grey dotted line indicates the cut-off for term birth (37 weeks GA or later is term-born). *** indicates $p < 0.001$, ** indicates $p < 0.01$, * indicates $p < 0.05$.

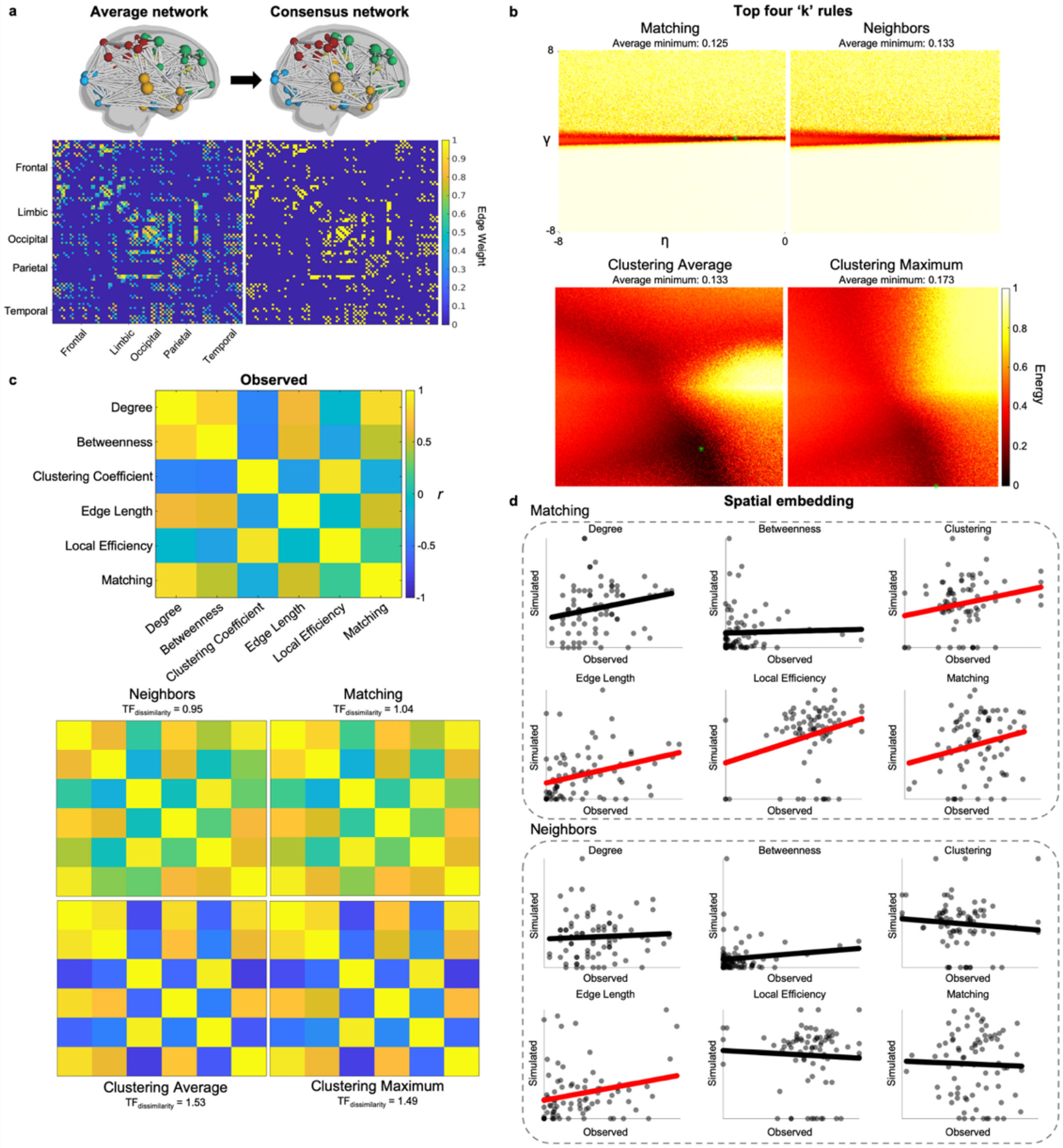

Supplementary Figure 8. 'Value' rule assessment through consensus network simulations. (a) Averaged weighted connectivity matrix compared to the binary matrix of the distant-dependent group representative consensus network. The node connectivity plots are adapted from AAL90 atlas in Shi F, et al. (2011) Infant Brain Atlases from Neonates to 1- and 2-Year-Olds. PLoS ONE 6(4): e18746. doi:10.1371/ journal.pone.0018746. **(b)** The average energy landscapes of the top four rules (based on lowest energies) are plotted with a green asterisk (*) indicating the location of the best fitting model. Homophily models performed the best out of all rules. 'Neighbors' had the lowest overall energy. 'Matching' had the lowest energy average over the top 10 models. **(c)** The topological fingerprint of the observed network compared to the top four performing 'value' rules. 'Neighbors' had the lowest topological dissimilarity, followed by 'matching'. **(d)** 'Matching' displayed spatial embedding for four local organization measures (degree: p = 0.050; betweenness: p = 0.787; clustering coefficient: $r_{pearson}$ = 0.23; p = 0.037; edge length: $r_{pearson}$ = 0.33; p = 0.003; local efficiency: $r_{pearson}$ = 0.30; p = 0.007; matching: $r_{pearson}$ = 0.23; p = 0.043). 'Neighbors' had significant spatial embedding for edge length ($r_{pearson}$ = 0.24, p = 0.035) but not on the other five local measures assessed (degree: p = 0.637; betweenness: p = 0.267; clustering coefficient: p = 0.400; local efficiency: p = 0.642; matching: p = 0.776). Red lines indicate significant correlations.

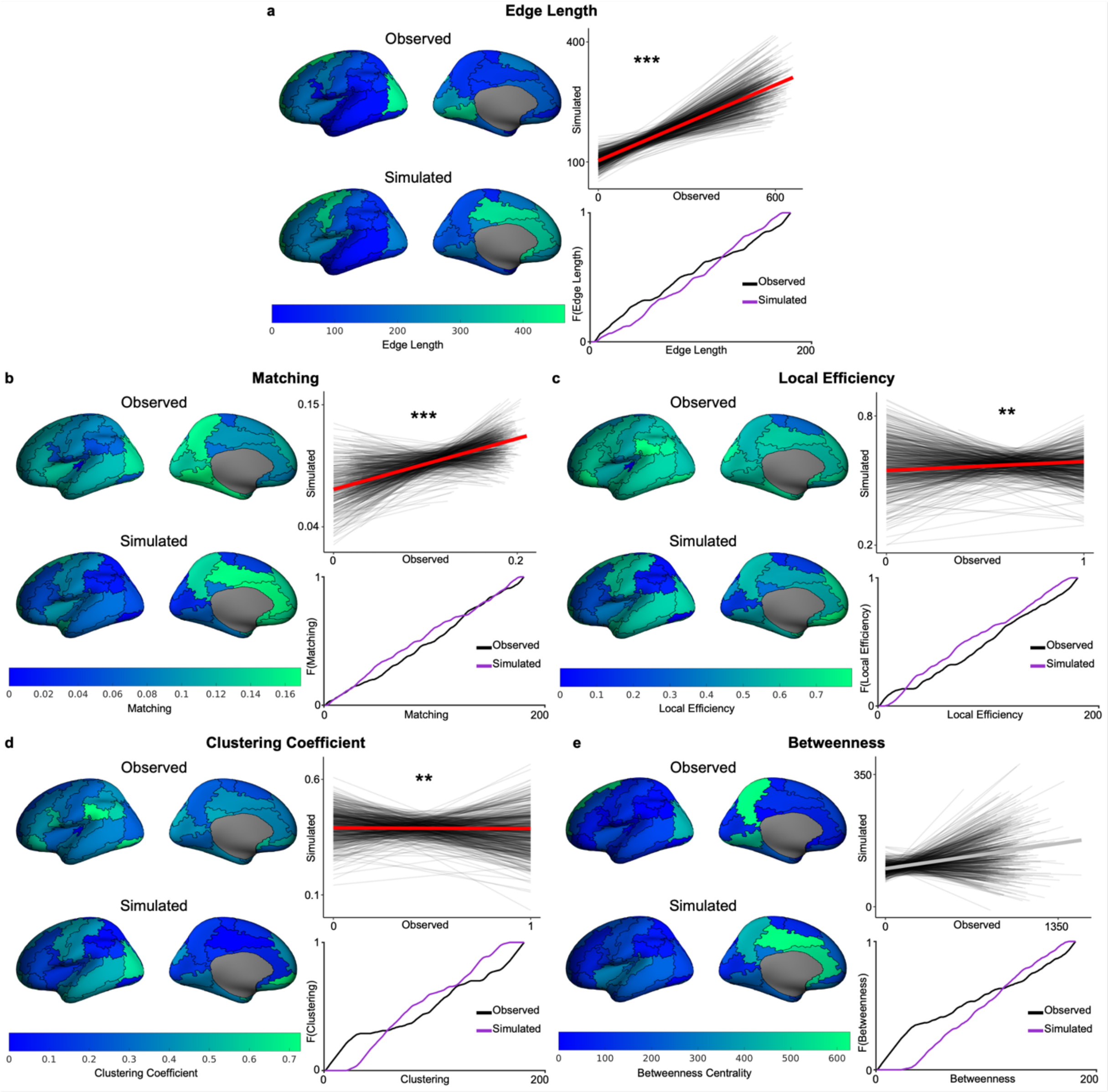

Supplementary Figure 9. Spatial embedding of optimized generative models. (a) Significant spatial embedding of local organization appears for edge length, **(b)** matching, **(c)** local efficiency, **(d)** clustering coefficient, **(e)** but not for betweenness. The top right graphs depict correlational data between observed and simulated measures in every region. A red line indicates a significant linear model, depicted as the average correlation and the grey lines show every participant's data. The bottom right graphs show the cumulative density function of the average observed (black) and simulated (purple) networks. *** indicates $p < 0.001$, ** indicates $p < 0.01$, * indicates $p < 0.05$. All surface plots are adapted from AAL90 atlas in Shi F, et al. (2011) Infant Brain Atlases from Neonates to 1- and 2-Year-Olds. PLoS ONE 6(4): e18746. doi:10.1371/ journal.pone.0018746.

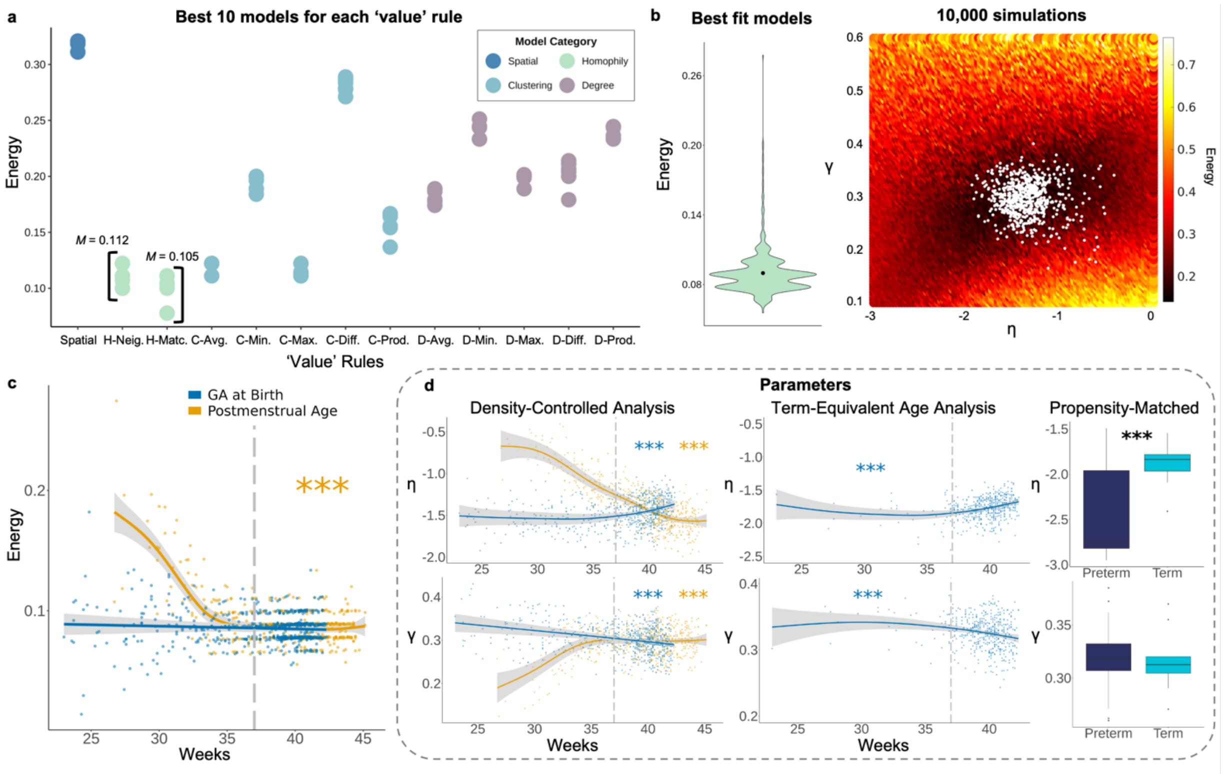

Supplementary Figure 10. Density-controlled analysis and parameter changes. (a) Energies from 90,000 simulations in -8 ≤ η ≤ 0 and -8 ≤ γ ≤ 8 sampling space fit to the density-controlled consensus network. The violin plot displays the lowest 1,000 energies for all 13 'value' rules. 'Matching' had the lowest overall energy (0.08) and lowest average (0.105) followed by 'neighbors' with a lowest energy of 0.10 and average of 0.112. **(b)** The average energy landscape of the density-controlled 'matching' models. The white dots indicate the location of the best-fit models for all participants. The violin plot demonstrates the disruption of energies of the best-fit models (maximum = 0.28, minimum = 0.06, *M* = 0.09, *SD* = 0.02). **(c)** Energy significantly decreased across PMA ($F_{energy,PMA}$ = 32.88, estimated df = 7.46, p = 2.00 x 10^{-16}) but not birth age (p = 0.380). **(d)** The density-controlled analysis replicates main manuscript with η significantly decreasing across PMA and increasing across GA at Birth ($F_{\eta,PMA}$ = 39.56, estimated df = 5.51, p = 2.00 x 10^{-16}; $F_{\eta,GA\ at\ birth}$ = 5.14, estimated df = 3.19, p = 4.06 x 10^{-4}) and γ significantly increasing across PMA and decreasing across GA at Birth ($F_{\gamma,PMA}$ = 11.34, estimated df = 5.18, p = 2.00 x 10^{-16}; $F_{\gamma,GA\ at\ birth}$ = 14.67, estimated df = 1.12, p = 5.50 x 10^{-5}). The term-equivalent age analysis is also consistent ($F_{\eta,GA\ at\ birth}$ = 7.29, estimated df = 3.06, p = 1.92 x 10^{-5}; $F_{\gamma,GA\ at\ birth}$ = 5.67, estimated df = 2.30, p = 8.42 x 10^{-4}). The propensity-matched analysis shows significantly lower eta (t(180.83) = -11.80, p = 2.20 x 10^{-16}) but not significantly higher gamma for the preterm group compared to the term group (p = 0.792). This suggests that differences in gamma at early PMA for preterm groups may resolve by term-equivalent age. The shaded area indicates 95% confidence intervals. The grey dotted line indicates the cut-off of term-born infants (37 weeks GA or later is term-born). *** indicates p < 0.001, ** indicates p < 0.01, * indicates p < 0.05.

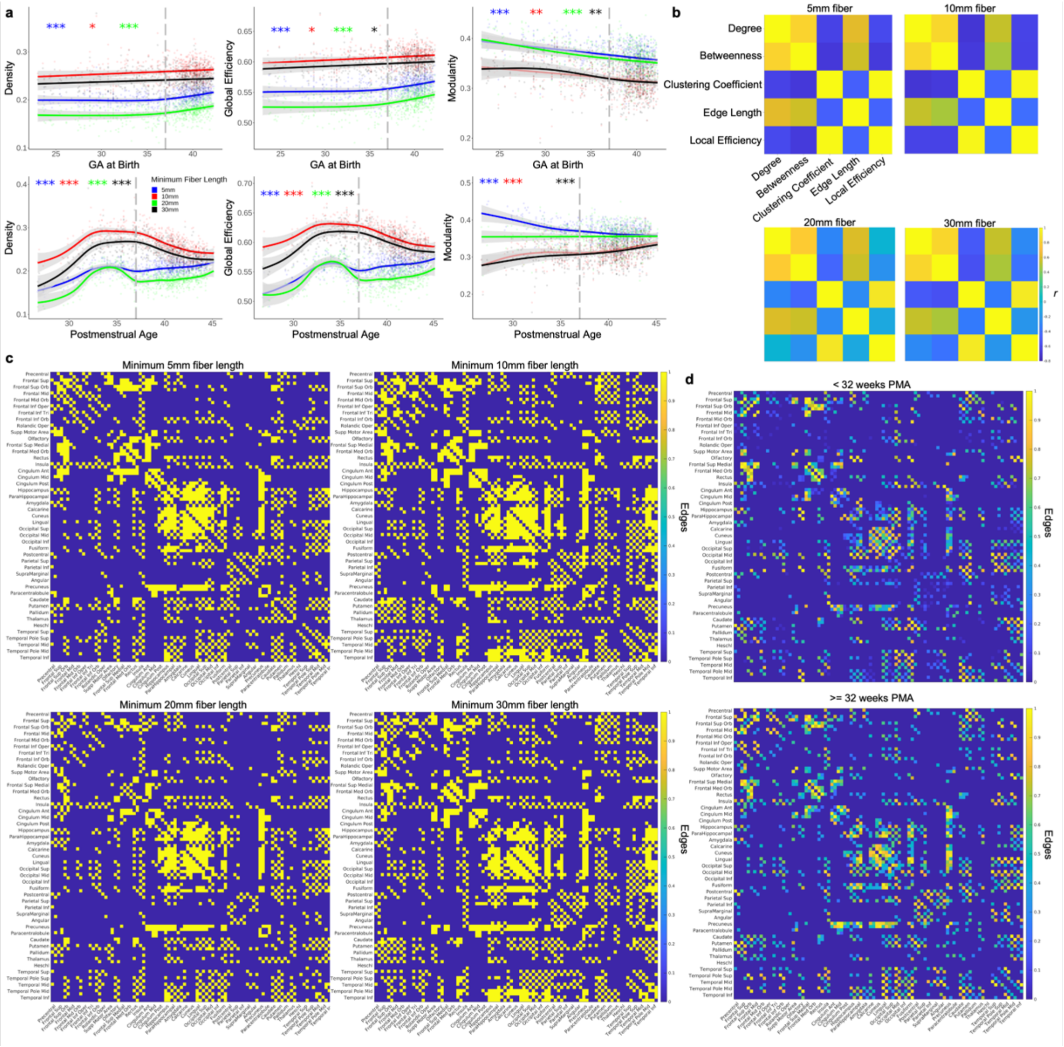

Supplementary Figure 11. Exploration of the effects of minimum fiber length on tractography. (a) Density (5mm*:* $F_{density,PMA}$ = 11.77, estimated df = 8.43, p = 2.00 x 10^{-16}, $F_{density,GA\ at\ birth}$ = 7.41, estimated df = 3.29, p = 6.18 x 10^{-6}; 10mm*:* $F_{density,PMA}$ = 39.71, estimated df = 7.71, p = 2.00 x 10^{-16}, $F_{density,GA\ at\ birth}$ = 4.23, estimated df = 1.00, p = 0.040; 20mm*:* $F_{density,PMA}$ = 15.98, estimated df = 10.85, p = 2.00 x 10^{-16}, $F_{density,GA\ at\ birth}$ = 6.22, estimated df = 2.80, p = 1.55 x 10^{-4}; 30mm*:* $F_{density,PMA}$ = 37.17, estimated df = 7.50, p = 2.00 x 10^{-16}, *GA at birth:* p = 0.166), global efficiency (5mm*:* $F_{global\ efficiency,PMA}$ = 10.89, estimated df = 8.69, p = 2.00 x 10^{-16}, $F_{global\ efficiency,GA\ at\ birth}$ = 7.06, estimated df = 2.93, p = 3.34 x 10^{-5}; 10mm*:* $F_{global\ efficiency,PMA}$ = 37.84, estimated df = 6.87, p = 2.00 x 10^{-16}, $F_{global\ efficiency,GA\ at\ birth}$ = 6.25, estimated df = 1.00, p = 0.013; 20mm*:* $F_{global\ efficiency,PMA}$ = 12.10, estimated df = 10.16, p = 2.00 x 10^{-16}, $F_{global\ efficiency,GA\ at\ birth}$ = 6.56, estimated df = 2.72, p = 1.08 x 10^{-4}; 30mm*:* $F_{global\ efficiency,PMA}$ = 35.29, estimated df = 7.23, p = 2.00 x 10^{-16}, $F_{global\ efficiency,GA\ at\ birth}$ = 4.21, estimated df = 1.00, p = 0.041), and modularity (5mm*:* $F_{modularity,PMA}$ = 6.99, estimated df = 3.51, p = 8.83 x 10^{-6}, $F_{modularity,GA\ at\ birth}$ = 19.98, estimated df = 1.00, p = 9.53 x 10^{-6}; 10mm*:* $F_{modularity,PMA}$ = 7.15, estimated df = 4.19, p = 1.43 x 10^{-6}, $F_{modularity,GA\ at\ birth}$ = 9.18, estimated df = 1.00, p = 2.53 x 10^{-3}; 20mm*: PMA* p = 0.900, $F_{modularity,GA\ at\ birth}$ = 10.32, estimated df = 1.86, p = 2.19 x 10^{-5}; 30mm*:* $F_{modularity,PMA}$ = 6.85, estimated df = 2.84, p = 7.64 x 10^{-5}, $F_{modularity,GA\ at\ birth}$ = 4.38, estimated df = 2.80, p = 3.46 x 10^{-3}) across GA at birth and PMA. **(b)** Topological fingerprints and **(c)** consensus networks for each tractography method. **(d)** Average binarized matrices of early and late PMA neonatal scans for 30mm fiber length connectomes. The shaded area indicates 95% confidence intervals. The grey dotted line indicates the cut-off of term-born infants (37 weeks GA or later is term-born). *** indicates $p < 0.001$, ** indicates $p < 0.01$, * indicates $p < 0.05$.

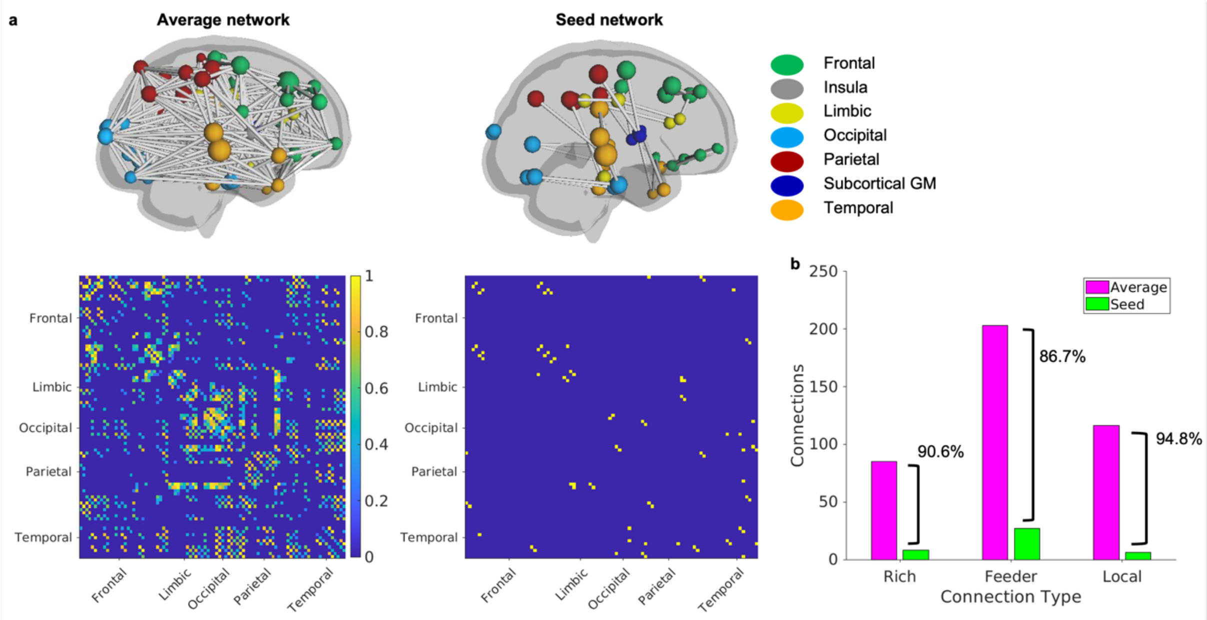

Supplementary Figure 12. Comparison of seed network for GNMs with average binarized network. (a) The average network across all participants and the sparse seed network calculated from edges present in 95% of all participants (total connections = 41). On average, the seed network is about 10% of the total connections for each individual (average total connections = 404.6). The node connectivity plots are adapted from AAL90 atlas in Shi F, et al. (2011) Infant Brain Atlases from Neonates to 1- and 2-Year-Olds. PLoS ONE 6(4): e18746. doi:10.1371/ journal.pone.0018746. **(b)** The number of connections, separated by connection type, on average across all participants and for the seed network.
