# Supplementary material for: Premature birth changes wiring constraints in neonatal structural brain networks
Source: Nat Commun. 2025 Jan 8;16:490. doi: 10.1038/s41467-024-55178-x (PMC11711473; doi:10.1038/s41467-024-55178-x)
Supplement: Supplementary file 2 — Reporting Summary [file 41467_2024_55178_MOESM2_ESM.pdf]

nature portfolio

Corresponding author(s): Alexa Mousley

Last updated by author(s): Oct 17, 2024

Reporting Summary

Nature Portfolio wishes to improve the reproducibility of the work that we publish. This form provides structure for consistency and transparency in reporting. For further information on Nature Portfolio policies, see our Editorial Policies and the Editorial Policy Checklist.

Statistics

For all statistical analyses, confirm that the following items are present in the figure legend, table legend, main text, or Methods section.

n/a	Confirmed	
☐	☒	The exact sample size (*n*) for each experimental group/condition, given as a discrete number and unit of measurement
☐	☒	A statement on whether measurements were taken from distinct samples or whether the same sample was measured repeatedly
☐	☒	The statistical test(s) used AND whether they are one- or two-sided<br>*Only common tests should be described solely by name; describe more complex techniques in the Methods section.*
☐	☒	A description of all covariates tested
☐	☒	A description of any assumptions or corrections, such as tests of normality and adjustment for multiple comparisons
☐	☒	A full description of the statistical parameters including central tendency (e.g. means) or other basic estimates (e.g. regression coefficient) AND variation (e.g. standard deviation) or associated estimates of uncertainty (e.g. confidence intervals)
☐	☒	For null hypothesis testing, the test statistic (e.g. *F*, *t*, *r*) with confidence intervals, effect sizes, degrees of freedom and *P* value noted<br>*Give P values as exact values whenever suitable.*
☒	☐	For Bayesian analysis, information on the choice of priors and Markov chain Monte Carlo settings
☐	☒	For hierarchical and complex designs, identification of the appropriate level for tests and full reporting of outcomes
☐	☒	Estimates of effect sizes (e.g. Cohen's *d*, Pearson's *r*), indicating how they were calculated

Our web collection on statistics for biologists contains articles on many of the points above.

Software and code

Policy information about availability of computer code

Data collection	No data was collected by the authors. All data used was originally from the Developing Human Connectome Project third data release (http://www.developingconnectome.org/data-release/third-data-release/). The data used in the study was accessed from DSI Studio in a semi-processed format (https://brain.labsolver.org/hcp_d2.html). All data derived in the study is public on Open Science Framework (https://osf.io/ng43c/).
Data analysis	All analysis scripts have been published on GitHub: https://github.com/alexamousley/neonatal_generative_network_modeling

For manuscripts utilizing custom algorithms or software that are central to the research but not yet described in published literature, software must be made available to editors and reviewers. We strongly encourage code deposition in a community repository (e.g. GitHub). See the Nature Portfolio guidelines for submitting code & software for further information.

Data

Policy information about availability of data

All manuscripts must include a data availability statement. This statement should provide the following information, where applicable:

- Accession codes, unique identifiers, or web links for publicly available datasets
- A description of any restrictions on data availability
- For clinical datasets or third party data, please ensure that the statement adheres to our policy

All derived, anonymized data used in this publication is available at: https://osf.io/ng43c/. The semi-processed version of dHCP data used in this publication is

available at: https://brain.labsolver.org/hcp_d2.html. The dHCP original data is available on their public repository: https://data.developingconnectome.org/app/template/Login.vm.

Research involving human participants, their data, or biological material

Policy information about studies with human participants or human data. See also policy information about sex, gender (identity/presentation), and sexual orientation and race, ethnicity and racism.

Reporting on sex and gender	Sex data (as a biological attribute) was accessed from dHCP dataset. Sex was not relevant to the research questions of interested in this is paper but was used as a covariate. No gender data was accessed.
Reporting on race, ethnicity, or other socially relevant groupings	No race, ethnicity or social data was accessed.
Population characteristics	No population characteristics were accessed.
Recruitment	Participants were recruit through the dHCP. Exclusion and inclusion information can be accessed here: http://www.developingconnectome.org/study-inclusion-and-exclusion-criteria/
Ethics oversight	The dHCP is funded through a Synergy Grant by the European Research Council (ERC) under the European Union's Seventh Framework Programme (FP/2007-2013) / ERC Grant Agreement n. 319456.

Note that full information on the approval of the study protocol must also be provided in the manuscript.

Field-specific reporting

Please select the one below that is the best fit for your research. If you are not sure, read the appropriate sections before making your selection.

☒ Life sciences ☐ Behavioural & social sciences ☐ Ecological, evolutionary & environmental sciences

For a reference copy of the document with all sections, see nature.com/documents/nr-reporting-summary-flat.pdf

Life sciences study design

All studies must disclose on these points even when the disclosure is negative.

Sample size	N = 758 was collected by dHCP. Dr. Yeh published N = 724 semi-processed scans after removing 34 participants for poor quality scan (identified with the neighboring DWI correlation method). We removed 94 of these scans for being marked as a repeat scan, resulting in a cross-sectional sample (N = 630).
Data exclusions	No data was excluded.
Replication	No studies to date use this method with this sample. Measures have been taken the maximize transparency for the sake of future reproducibility. For example, all code and example data to implement scripts are public here: https://github.com/alexamousley/neonatal_generative_network_modeling
Randomization	No randomization was required as the variable of interest, being a term or preterm infant, is a characteristic of participants.
Blinding	No blinding was required.

Reporting for specific materials, systems and methods

We require information from authors about some types of materials, experimental systems and methods used in many studies. Here, indicate whether each material, system or method listed is relevant to your study. If you are not sure if a list item applies to your research, read the appropriate section before selecting a response.

Materials & experimental systems

n/a	Involved in the study
☒	☐ Antibodies
☒	☐ Eukaryotic cell lines
☒	☐ Palaeontology and archaeology
☒	☐ Animals and other organisms
☒	☐ Clinical data
☒	☐ Dual use research of concern
☒	☐ Plants

Methods

n/a	Involved in the study
☒	☐ ChIP-seq
☒	☐ Flow cytometry
☐	☒ MRI-based neuroimaging

Plants

Seed stocks: N/A

Novel plant genotypes: N/A

Authentication: N/A

Magnetic resonance imaging

Experimental design

Design type: Diffusion MRI

Design specifications: Duration of dMRI acquisition: 19:20

Behavioral performance measures: No behavioral tasks were accessed

Acquisition

Imaging type(s): Diffusion

Field strength: 3T Philips Achieva

Sequence & imaging parameters: EPI sequence were used (MB factor 4, SENSE factor 1.2, partial Fourier factor 0.86, in-plane resolution 1.5 × 1.5 mm, 3 mm slices with 1.5 mm overlap, TE = 90 ms, TR = 3800 ms). For more information, see: Edwards, A. D., Rueckert, D., Smith, S. M., Abo Seada, S., Alansary, A., Almalbis, J., ... & Hajnal, J. V. (2022). The developing human connectome project neonatal data release. Frontiers in neuroscience, 16, 886772.

Area of acquisition: Whole-brain scan

Diffusion MRI: ☒ Used ☐ Not used

Parameters: dMRI was performed in 4 shells (b = 0 s/mm2: 20, b = 400 s/mm2: 64, b = 1000 s/mm2: 88, b = 2600 s/mm2: 128). Each shell was split into 4 subsets acquired using AP, PA, RL, and LR phase encoding. For more information, see: Edwards, A. D., Rueckert, D., Smith, S. M., Abo Seada, S., Alansary, A., Almalbis, J., ... & Hajnal, J. V. (2022). The developing human connectome project neonatal data release. Frontiers in neuroscience, 16, 886772.

Preprocessing

Preprocessing software: Authors accessed data in a preprocessed format. For information about preprocessing methods, please see: https://brain.labsolver.org/hcp_d2.html and https://biomedia.github.io/dHCP-release-notes/dwi-shard.html

Normalization: Authors accessed data in a preprocessed format. The dHCP SHARD pipeline preformes normalised mutual information (NMI) based registration with FSL Flirt on the mean b=1000 s/mm2 shell. For more information, please see: https://biomedia.github.io/dHCP-release-notes/dwi-shard.html

Normalization template: dHCP 40 week template. For more information, please see: Schuh, A., Makropoulos, A., Robinson, E. C., Cordero-Grande, L., Hughes, E., Hutter, J., ... & Rueckert, D. (2018). Unbiased construction of a temporally consistent morphological atlas of

neonatal brain development. BioRxiv, 251512.

Noise and artifact removal

Authors accessed data in a preprocessed format. For information about noise and artifact removal, please see: https://brain.labsolver.org/hcp_d2.html and https://biomedia.github.io/dHCP-release-notes/dwi-shard.html

Volume censoring

No volume censoring was preformed by the authors.

Statistical modeling & inference

Model type and settings

Generalized additive models (GAMs) with penalized splines were performed with the mgcv package in R v4.1.2. Models were fit with the restricted maximum likelihood (REML) method. Cubic regression splines were used as the basis function. The curvature of the model was assessed by fitting the model with cubic shrinkage splines. Postmenstrual age, sex, head circumference, translation, and rotation were controlled for in GAMs on observed data.

Effect(s) tested

We explored the effect of gestational age at birth and postmenstrual age.

Specify type of analysis: ☐ Whole brain ☒ ROI-based ☐ Both

Anatomical location(s)

We used the AAL90 atlas: Shi, F. et al. Infant brain atlases from neonates to 1- and 2-year-olds. PLoS One 6, e18746 (2011).

Statistic type for inference

(See Eklund et al. 2016)

We used a 90 parcellation to explore macro-scale organization.

Correction

For local statistics, we used the Benjamini-Hochberg false discovery rate correction: Benjamini, Y. & Hochberg, Y. Controlling the false discovery rate: A practical and powerful approach to multiple testing. J. R. Stat. Soc. 57, 289–300 (1995).

Models & analysis

n/a	Involved in the study
☒	☐ Functional and/or effective connectivity
☐	☒ Graph analysis
☐	☒ Multivariate modeling or predictive analysis

Graph analysis

We used subject-level binarized graphs.

Multivariate modeling and predictive analysis

This analysis used birth and postmenstrual age to predict topology. The main text focused on global measures, using local measures solely to look at model fitting.

Local statistics : degree, betweenness, clustering coefficient, edge length, local efficiency, and matching.
Global statistics: maximum modularity, characteristic path length, global efficiency, connection types (rich, feeder and local) and connection lengths (rich, feeder and local).
